# Prevalence and Drug Resistance of Nontuberculous Mycobacteria, Northern China, 2008–2011

**DOI:** 10.3201/eid2007.131801

**Published:** 2014-07

**Authors:** Xiaobo Wang, Hao Li, Guanglu Jiang, Liping Zhao, Yifeng Ma, Babak Javid, Hairong Huang

**Affiliations:** Beijing Tuberculosis and Thoracic Tumor Institute, Beijing, China (X. Wang, G. Jiang, L. Zhao, Y. Ma, H. Huang);; Tsinghua University, Beijing (H. Li, B. Javid);; Collaborative Innovation Centre for Diagnosis and Treatment of Infectious Disease, Hangzhou, China (B. Javid)

**Keywords:** Mycobacteria tuberculosis, nontuberculous, bacteria, mycobacteria, China, Mycobacteriaceae, antibiotic, antimicrobial, resistance

**To the Editor:** Nontuberculous mycobacteria (NTM), defined as members of *Mycobacterium* species other than those in the *M. tuberculosis* complex or *M. leprae*, are mostly considered to be opportunistic pathogens (*1*). However, many NTM can and do cause disease in immune-competent hosts. Pulmonary infection by NTM can be a source of diagnostic uncertainty, especially in locations such as in China, where acid-fast staining of sputum samples is the mainstay of diagnosis for tuberculosis (*2*). NTM are also relatively resistant to many of the first- and second-line drugs used to treat tuberculosis, thus making accurate diagnosis and drug-susceptibility testing critical to clinical management of NTM infections (*3*). The medical and public health communities have been concerned about increasing prevalence of NTM infection in China, and 2 recent surveys, 1 from Shanghai and another from a rural population in Shandong Province, gave somewhat conflicting reports of the prevalence of these infections (*4*,*5*). We therefore decided to conduct a survey of NTM isolates in Beijing from the National Tuberculosis Clinical Laboratory of the Beijing Chest Hospital. We also tested isolates from specimens collected in this laboratory against an extended drug susceptibility panel to determine which drug regimens would be most useful in therapy for various NTM infections.

During January 2008–December 2011, sputum samples collected from 3,714 patients attending the Beijing Chest Hospital with suspected pulmonary tuberculosis were positive for mycobacterial spp. Among the surveillance population, 92% were from northern China, including 13 provinces and the 2 major urban conurbations of Beijing and Tianjin. From our survey, the Han ethnic group accounted for 82% of patients, and 61% of total patients were from urban, rather than rural, areas. Most (59%) of the patients were male, and 40% were attending the hospital for re-treatment of pulmonary tuberculosis; mean age was 51 ± 20 years. Of these mycobacterial isolates, 95 (2.6%) were positive for NTM; NTM were identified during initial screening for resistance to *p*-nitrobenzoic acid. We identified the strains to species level by sequencing the internal transcribed spacer region of the 16S-23S rRNA and 16S rRNA genes (*6*), which is able to discriminate between even closely related species such as *M. chelonae* and *M. abscessus* (*7*).

Of the 95 NTM isolates, 38 (40%) were *M. intracellulare* and 28 (29%) were *M. abscessus* (Table). Five additional species were also identified: *M. fortuitum* (8%), *M. gordonae* (8%), *M. kansasii* (7%), *M. avium* (5%), and *M. parascrofulaceum* (1%). A survey performed recently in Shandong Province also identified *M. intracellulare* as the most common isolate (*4*), but in that study, it represented 52 (81%) of 64 cases. By contrast, 2 previous surveys found *M. chelonae* to be the most commonly isolated species (20% and 27% of isolates) (*5*,*8*). However, none of the isolates from our study were *M. chelonae*. Differences in isolates may represent the representative patient population from which they were derived; *M. chelonae* was most commonly isolated from hospitals in southern China (*5*,*8*). The most common NTM species found in eastern Asia was *M. avium* complex, in keeping with findings from our study (*9*). Documenting another trend, the International Union Against Tuberculosis and Lung Disease reported that *M. fortuitum* was the most frequently encountered species in Turkey (33.9%), the Czech Republic (17.5%), Portugal (16.5%), and other countries in Europe (*10*).

**Table:** Species and drug-resistance profiles of 95 nontuberculous mycobacteria strains, northern China, 2008–2011*

Drugs	No. (%) resistant strains in *Mycobacterium* spp.							Total
	*M. intracellulare*	*M.abscessus*	*M.fortuitum*	*M. gordonae*	*M.kansassi*	*M. avium*	*M.parascrofulaceum*	
INH	37 (97.37)	28 (100)	7 (87.5)	6 (75)	3 (42.86)	5 (100)	1 (100)	87 (91.58)
RIF	34 (89.47)	28 (100)	7 (87.5)	2 (25)	0	5 (100)	1 (100)	77 (81.05)
EMB	4 (10.53)	26 (92.86)	7 (87.5)	1 (12.5)	0	2 (40)	0	40 (42.11)
SM	38 (100)	28 (100)	7 (87.5)	4 (50)	6 (85.71)	5 (100)	1 (100)	89 (93.68)
CPM	31 (81.58)	26 (92.86)	4 (50)	1 (12.5)	2 (28.57)	3 (60)	1 (100)	68 (71.58)
AK	31 (81.58)	25 (89.29)	4 (50)	1 (12.5)	1 (14.29)	4 (80)	0	66 (69.43)
PTO	25 (65.79)	27 (96.43)	6 (75)	4 (50)	0	4 (80)	1 (100)	67 (70.53)
PAS	38 (100)	28 (100)	7 (87.5)	8 (100)	7 (100)	4 (80)	1 (100)	93 (97.89)
OFLX	38 (100)	28 (100)	3 (37.5)	3 (37.5)	1 (14.29)	5 (100)	1 (100)	79 (83.16)
LVFX	36 (94.74)	28 (100)	3 (37.5)	2 (25)	0	5 (100)	1 (100)	75 (78.95)
Total	38 (40)	28 (29.47)	8 (8.42)	8 (8.42)	7 (7.37)	5 (5.26)	1 (1.05)	95 (100)

*INH, isoniazid; RIF, rifampin; EMB, ethambutol; SM, streptomycin; CPM, capreomycin; AK, amikacin; PTO, protionamide; PAS, para-aminosalicylic acid; OFLX, ofloxacin; LVFX, levofloxacin.

Drug susceptibility testing (DST) was performed by the proportion method according to the WHO Guidelines for the Programmatic Management of Drug-resistant Tuberculosis, 2011 Update [cited 2014 May 12] http://whqlibdoc.who.int/publications/2011/9789241501583_eng.pdf. We tested 3 first-line anti-tuberculosis drugs (rifampin, isoniazid, and ethambutol) and 7 second-line agents (streptomycin, capreomycin, amikacin, protionamide, para-amino salicylic acid, ofloxacin, and levofloxacin) (Table). If a patient had multiple positive NTM isolates, DST was performed on the last isolate. In agreement with other studies (*4*,*5*), ethambutol remained the most useful agent against NTM; its overall resistance rate among isolates tested was 42%. Ranking of second or third agents, however, should be guided by species identification and DST. For example, levofloxacin appears to be a good choice for *M. kansasii, M. gordonae*, or *M. fortiutum* infections (overall resistance rate 22%), but a poor choice against *M. avium* complex infections (overall resistance rate 95%). The second most prevalent species in our study (28% of isolates), *M. abscessus*, was resistant to the test drugs in >90% of cases, highlighting the difficulties associated with treatment for some NTM infections.

Our study suggests that there has been no substantial increase in the prevalence of NTM in respiratory isolates from persons in northern China. Most of the isolates show substantial and extensive drug resistance, providing major therapeutic challenges for clinicians, especially if patients are treated as they would be for drug susceptible tuberculosis. To guide therapy, both species-level identification and DST of NTM isolates should be performed. Our data suggest that testing the efficacy of some second-line agents, in particular, fluoroquinolones, may be beneficial in identifying further options for therapy.
